# Anxiety Disorder and Smoking Behavior: The Moderating Effects of Entertainment and Informational Television Viewing

**DOI:** 10.3390/ijerph19159160

**Published:** 2022-07-27

**Authors:** Juwon Hwang, Porismita Borah

**Affiliations:** 1 School of Media and Strategic Communications, Oklahoma State University, Stillwater, OK 74078, USA; 2 Edward R. Murrow College of Communication, Washington State University, Pullman, WA 99163, USA; p.borah@wsu.edu

**Keywords:** smoking, anxiety disorder, mood adjustment theory, television viewing, television genres, entertainment television, information television

## Abstract

Smoking is more common among individuals with mental health issues than those who do not have mental illnesses. In particular, among individuals with an anxiety disorder, a high prevalence of smoking has been found. Mood adjustment theory suggests that individuals with negative moods could adjust their moods depending on the type of television they watched. To understand this relationship better, we aim to examine how different television viewing can moderate the tendency of smoking behavior for individuals with an anxiety disorder. We used national U.S. survey data and concepts from the mood adjustment theory to answer our research questions. Our main contributions were to: (1) extend the mood adjustment theory by focusing on the association between a diagnosed mental disorder (i.e., anxiety) and risky behavior (i.e., smoking), and (2) examine the nuances of television genres by dividing entertainment television into excitement-valenced and ambiguously-valenced entertainment programs, along with information programs. The primary findings show that individuals with an anxiety disorder were more likely to smoke and this association was significantly attenuated when they watched cartoons, sports, and health information programs, but the positive association between an anxiety disorder and the extent of smoking was intensified when they watched drama, music, sci-fi, and television news. Patients with an anxiety disorder may take advantage of excitement-valenced entertainment programs and health-related information but need to be cautious in choosing ambiguously-valenced entertainment programs and news.

## 1. Introduction

According to statistics from the Centers for Disease Control and Prevention (CDC), cigarette smoking is the leading cause of preventable death in the United States. Smoking causes more than 480,000 deaths every year in the U.S. [1]. Multiple studies have linked mental health issues with risky behaviors, such as smoking (e.g., [2,3,4]). A study based on a U.S. national survey reported that tobacco use among patients with mental illness is higher compared to those without mental issues [2]. In particular, among patients diagnosed with an anxiety disorder, a high prevalence of smoking has been demonstrated [5,6].

Research shows that one way to adjust the tendency that patients with an anxiety disorder engage in smoking behavior is viewing television. Given that patients with mental illnesses could use television to displace their negative thoughts [7], it is likely that the act of viewing television ameliorates levels of anxiety, which, in turn, reduces engagement in risky behaviors, such as smoking. However, there is a myriad of content ranging from joyful to sad on television; thus, not all television genres may help patients with an anxiety disorder reduce the likelihood of engaging in smoking behavior. Built on the mood management theory, the present study extends the literature by focusing on: (1) patients with an anxiety disorder and (2) their risky behavior, as well as (3) differentiating entertainment television programs into excitement-valenced and ambiguously-valenced programs.

### 1.1. An Anxiety Disorder and Smoking Behavior

Risky behavior has been defined as “any consciously, or non-consciously controlled behavior with a perceived uncertainty about its outcome, and/or about its possible benefits or costs for the physical, economic or psycho-social well-being of oneself or others” ([8], p. 9). Statistics show that smoking is the primary cause of preventable deaths [1]. Empirical studies indicate that mental illnesses such as anxiety disorders are associated with nicotine dependence, low rates of smoking cessation, and withdrawal symptoms when quitting smoking [3,9]. Studies have shown that smoking is more common among patients with anxiety disorders compared to those who do not have anxiety disorders [5,6]. Research also demonstrates that mental issues such as anxiety disorders play an important role “in the initiation, maintenance, and cessation of smoking behavior” ([6], p. 283). Based on the literature, we begin with a replication hypothesis that postulates an association between an anxiety disorder and smoking behavior:

**H1.**  *Patients with an anxiety disorder are more likely to engage in smoking behavior compared to those who do not have an anxiety disorder.*

### 1.2. Theoretical Background: Mood Management Theory

Mood management theory suggests that individuals suffering from negative affective states are motivated to alter their current mood by choosing television content that maximizes pleasure and minimizes pain [10,11,12]. Pleasant and up-bringing entertainment content may enhance negative moods, whereas saddening or down-bringing entertainment content may intensify an adverse state [13,14]. Based on this theory, a wealth of evidence shows that people choose entertainment media to assist in regulating their affective states [13,14]. 

Although this theory has been predominantly applied to people who are assumed to be mentally healthy and capable of effective regulation of their moods [15], little attention has been paid to patients with mental illnesses. Patients with mental illnesses are, however, considered to be in extremely negative moods. Specifically, anxiety is a future-oriented mood disorder associated with preparation for possible, upcoming negative events [16]. Thus, it is important to focus on patient with an anxiety disorder in order to provide practical implications to this group of people with regard to the potential ways of preventing them from being buried in their affective states.

As noted above, the previous studies based on this theory have primarily focused on how the content of media alleviates negative moods, rather than behaviors. For example, participants in the negative mood condition spent significantly more time listening to energetic and joyful music, which helped them displace their bad moods with positive moods [17]. Similarly, anger in female participants was dissipated through exposure to positive news [18]. Despite substantial evidence showing that individuals with negative moods may alter their mood through media choice (e.g., [17,18]), to the best of our knowledge, no study has examined behavioral change with this approach. However, moods have the power to alter individuals’ behavioral responses to many objects and events [19]. If individuals’ negative moods are altered by their media choice as postulated in this theory, we contend that these changes may be reasonably represented in their behaviors as well. 

A body of literature focused on the effects of entertainment television viewing, since entertainment television genre undoubtedly reduces or terminates aversive moods through its positive hedonic valence [10,11,12]. However, these results are not consistent [20]. For example, comedy and drama could have different impact on individuals, even though they fall within the entertainment genre. Thus, it is important to offer more nuanced information about the roles of entertainment television viewing by moving beyond oversimplified categories (i.e., entertainment genre). This study divides entertainment television into excitement-valenced entertainment television (i.e., its content may be joyful, cheerful, and playful, such as comedy) and ambiguously-valenced entertainment television (i.e., its content may vary from sadness, horror, to happiness, such as movies and drama) as follows. 

#### 1.2.1. Excitement-Valenced Entertainment Television Programs

The literature shows that individuals who are experiencing negative affects should gravitate toward entertainment television genres such as comedy that have a positive valence in order to break out of their bad moods [20]. This implies that entertainment programs that serve in the regulation of moods should commonly have exciting, cheerful, and uplifting content. Thus, this study categorizes comedy, cartoons, and sports television programs into an excitement-valenced entertainment genre. 

The idea that links negative moods and excitement-valenced entertainment programs has indirectly been evident in numerous studies (e.g., [7,21]). For example, individuals experiencing stressful life events tended to watch comedy programs [7]. This suggests that patients with an anxiety disorder may benefit from excitement-valenced entertainment media. Moreover, as aforementioned, we focus on the moderating roles of television programs on the association between patients with an anxiety disorder and their smoking behavior. With the help of this literature, we hypothesize that excitement-valenced entertainment viewing would moderate the relationship between an anxiety disorder and smoking behavior in a way as follows:

**H2.**  *The association between an anxiety disorder and smoking behavior would be moderated by excitement-valenced entertainment television viewing, such that the strength of the positive association between an anxiety disorder and smoking would be reduced when they watched excitement-valenced entertainment television programs.*

#### 1.2.2. Ambiguously-Valenced Entertainment Television Programs

In contrast to excitement-valenced entertainment programs, several entertainment genres such as drama and movies have subgenres that deviate from what would normally be considered happy and uplifting [20]. Specifically, crime-laden drama, movies dwelling on violence, and sad songs are exemplary subgenres [20] that may interfere in the application of the mood management theory on the whole entertainment genre. At the same time, there are a fair amount of subgenres of these entertainment programs, such as comedy-mixed dramas that do not contain serious hardship but happy content that may be enough to uplift bad moods [22]. Thus, we call this type of entertainment genre, including drama and movies, an ambiguously-valenced entertainment genre, since its content that can be different depending on each program, including horror movies, comedy-mixed drama, and sad music. 

Compared to the clear explanation that individuals with negative moods improve their affective states with the help of excitement-valenced entertainment television such as comedy, prior research on mood management theory has been inconsistent in explaining how ambiguously-valenced entertainment programs impact individuals’ negative moods. Past literature shows that sad individuals exerted an increased interest in dramatic movies with serious themes of death [22] and gravitated to serious films and crime dramas but avoid dramatic comedies [23]. Similarly, people who feel nervous are likely to choose horror movies [23]. This seemingly odd preference for negatively-valenced entertainment content may occur because the content may satisfy individuals’ long-term hedonic goals because of its features of justice restoration or justification of one’s own level of aggressiveness [11]. Another body of research has explained that intense and dramatic plots featuring sadness, suffering, and pain may accentuate their existing negative moods, rather than regulating their moods, challenging the basic assumption of the mood management approach. For example, sad content dampens their spirits and pleasant feelings from media content accentuate individuals’ positive moods [19]. 

Music, one of the ambiguously-valenced entertainment genres, also produces inconsistent findings on the effects on terminating bad moods. On the one hand, people may select uplifting music to improve their negative mood states [17]. On the other hand, it is shown that participants in a sad mood chose songs that were sad and slow in tempo to continue feeling sad and gain insight into understanding tragedy [24]. Based on this inconsistent evidence, we expect that unhappy content included in ambiguously-valenced entertainment media offerings may or may not ameliorate the level of anxiety. Thus, we propose a research question to investigate the role of ambiguously-valenced entertainment content in reducing smoking behavior among patients with an anxiety disorder.

**RQ1.**  *What role does ambiguously-valenced entertainment television viewing play in moderating the association between an anxiety disorder and smoking behavior?*

#### 1.2.3. Informational Television Programs

The impact of information television with respect to its mood management power is clearly different from the one of entertainment television [10,25]. With regard to exposure to informational offerings, individuals consciously focus on the content of high educational value, which helps them to deal with life more effectively in the future [25]. In other words, the desire to learn something about the self and to receive information that aids in improving the current situation drives people to non-hedonistic media content, such as news and information programs [26]. As a result, the usage of informational programs may improve individuals’ affective, cognitive, and behavioral outcomes [7] because educational information in these programs helps individuals in bad moods cope with challenges more effectively. However, the benefits of informational offerings may vary depending on the extent to which the content of these offerings directly helps improve specific consequences. For example, health informational programs may play an active role in guiding viewers’ illnesses, symptoms, and overall health, by providing educational information. Thus, it is possible that health information programs mitigate the possibility of risk behaviors such as poor diet, smoking, and less involvement in disease prevention [27]. 

Next, research on the roles of television news has been inconsistent. For example, news and documentary programs may have dysphoric content that is related to stressful events in daily lives [7]. Thus, it is possible that news programs do not help mood management. However, since news is one of the educational television programs, it is believed that news may improve individuals’ affective, cognitive, and behavioral outcomes [7,26]. In sum, different programs within informational television could have distinct effects on individuals’ moods, including anxiety levels. To examine this relationship, we pose our last research question:

**RQ2.**  *What role does informational television viewing play in moderating the association between an anxiety disorder and smoking behavior?*

## 2. Methods

### 2.1. Data

The data for the present study came from the Multimedia Audience Research Systems (MARS) over the counter (OTC)/direct to consumer (DTC) Pharmaceutical Study of 2013 by Kantar Media. The MARS study investigated individuals’ patterns of OTC/DTC drugs consumption, health conditions, health-related behaviors, and health information use. The MARS data were collected from January 2013 to April 2013.

### 2.2. Sample

The MARS study consisted of a national sample and a list-enhanced oversample. The national sample was obtained through a systematic random sampling procedure from KBM’s AmeriLink database of 242 million consumers in the U.S. The list-enhanced sample was included to collect more information about people with various ailments such as asthma, diabetes, and/or high blood pressure within the past 24 months. The MARS data selected 48,666 potential participants with an expected response rate of 50% and questionnaire packets were sent to them. A total of 19,420 participants completed and returned questionnaire packets, for a response rate of 42.8%.

### 2.3. Measures

#### 2.3.1. Smoking Behavior

Respondents were asked a single-item question about the amount of cigarette packs being smoked in the last 7 days on a 5-point scale (0 = 0, less than 1 = 1, 1 to 4 = 2, 5 to 8 = 3, 9 or more = 4) (M = 0.39, SD = 0.95).

#### 2.3.2. Anxiety Disorder

The presence or absence of an anxiety disorder was assessed in the questionnaire on a binary scale (yes = 1, no = 0) by asking respondents whether a doctor or other health professional had given them a diagnosis of anxiety in the last 12 months (N = 1292, 6.7%). 

#### 2.3.3. Television Genres

Respondents were asked to indicate the types of television programs they watched in the last 7 days on a binary scale. Television program genres included news (N = 13,328, 68.6%), health information (N = 2185, 11.3%), situational comedy (N = 7027, 36.2%), cartoon (N = 2607, 13.4%), sports (N = 8847, 45.6%), drama (N = 9325, 48%), movie (N = 13,161, 67.8%), music (N = 4231, 21.3%), and sci-fi/fantasy (N = 3871, 19.9%). 

#### 2.3.4. Demographics, Stress Levels, and Total Hours per Week Spent Watching Television

Gender, age, race, education, and household income were included as control variables in the analysis. Gender was recorded as binary with 1 being male (30.7%) and 2 being female (69.3%). Respondents were asked to report their age using 13 ordinal categories and they were grouped into one of 13 age groups: 18–20 (0.8%), 21–24 (2.3%), 25–29 (4.3%), 30–34 (6.6%), 35–39 (5.9%), 40–44 (7.0%), 45–49 (8.0%), 50–54 (11.2%), 55–59 (11.4%), 60–64 (10.9%), 65–69 (10.0%), 70–74 (8.6%), or 75 or more (13.2%). Race was recorded with 1 being Caucasian (86.9%) and 0 being others (13.1%). Education was categorized into 4 categories: less than high school (5.8%), high school graduate (29.5%), some college (30.4%), and college graduate or more (34.4%). A household income variable was assessed using 10 increasing income ranges, and it was collapsed into 3 categories: less than $20,000 (42.8%), $20,000–$49,999 (41.3%), and $50,000 or more (15.9%). Finally, since we focus on the moderating roles of television genres, we also controlled for total hours per week spent watching television (M = 15.39, SD = 4.14). 

### 2.4. Analytic Strategy

Analysis of covariance (ANCOVA) was performed to compare the extent of smoking between individuals with and without anxiety, while controlling for demographics, stress levels, and total hours per week spent watching television. Next, a hierarchical linear regression analysis was performed to examine the moderating role of a set of television genres on the associations between an anxiety disorder and smoking behavior. The analysis was performed in three steps: smoking behavior was entered as a continuous dependent variable; control variables were entered in Step 1; an anxiety disorder as an independent variable and television viewing with nine sets of genres as moderators were entered in Step 2; and finally, the interactions between an anxiety disorder and each television genre were entered in Step 3. The analyses were conducted using SPSS version 21.

## 3. Results

### 3.1. The Association between an Anxiety Disorder and Risky Behavior

Hypothesis 1 predicted that patients with an anxiety disorder would show a greater amount of smoking behavior. The ANCOVA revealed that patients with an anxiety disorder were more likely to engage in smoking behavior (M = 0.79, SD = 1.25) than those without anxiety (M = 0.36, SD = 0.92), after controlling for six covariates, F(1, 19,412) = 132.506, *p* < 0.001. This finding was robust, emerging with a linear regression model, which was designed for responding to H2 and later. The regression model also showed that, while accounting for control variables, patients with an anxiety disorder tended to smoke more than those without anxiety (*b* = 0.210, *p* = 0.002) (see Table 1).

### 3.2. The Moderating Roles of Excitement-Valenced Entertainment Television Genres in the Association between an Anxiety Disorder and Smoking

**Figure 1:**
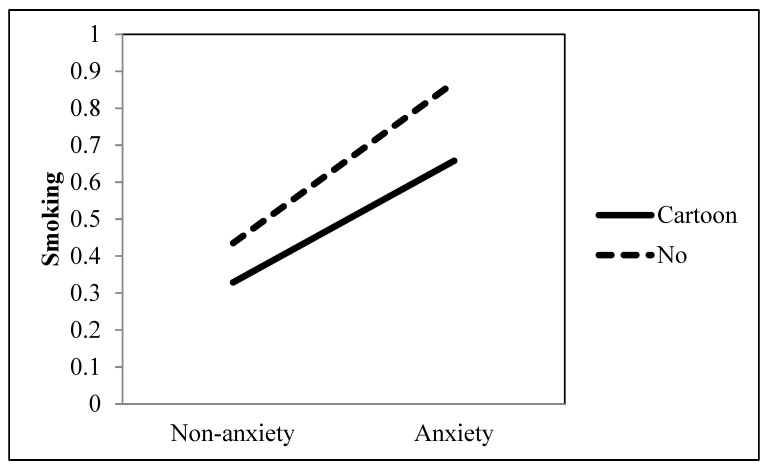
Interaction between anxiety and cartoon as a TV genre on smoking behavior.

**Figure 2:**
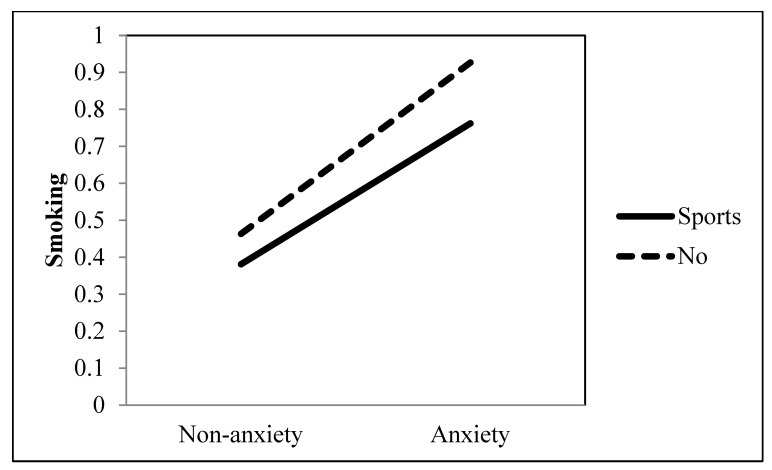
Interaction between anxiety and sports as a TV genre on smoking behavior.

Hypothesis 2 predicted that the association between an anxiety disorder and the extent of smoking would be moderated by excitement-valenced entertainment television viewing, such that the effect of the positive association would be reduced when they watched excitement-valenced entertainment programs including comedy, cartoon, and sports. The regression analysis demonstrated that the effect of the positive association between an anxiety disorder and smoking was significantly attenuated when they watched cartoons (*b* = −0.170, *p* = 0.009) and sports (*b* = −0.124, *p* = 0.025), but not comedy (*b* = 0.090, *p* = 0.104) (see Table 1 and Figure 1 and Figure 2). Thus, H2 was partially supported.

### 3.3. The Moderating Roles of Ambiguously-Valenced Entertainment Television Genres in the Association between an Anxiety Disorder and Smoking

**Figure 3:**
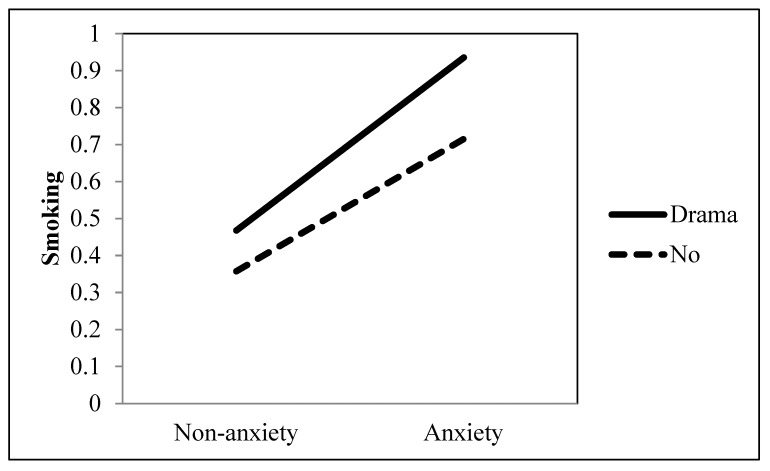
Interaction between anxiety and drama as a TV genre on smoking behavior.

**Figure 4:**
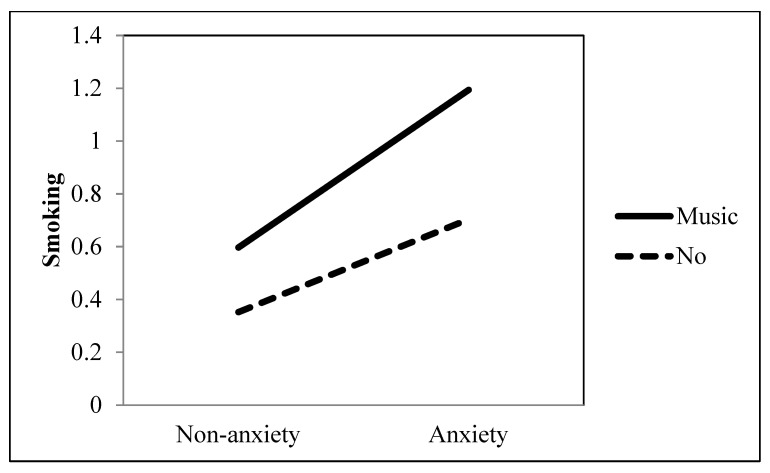
Interaction between anxiety and music as a TV genre on smoking behavior.

**Figure 5:**
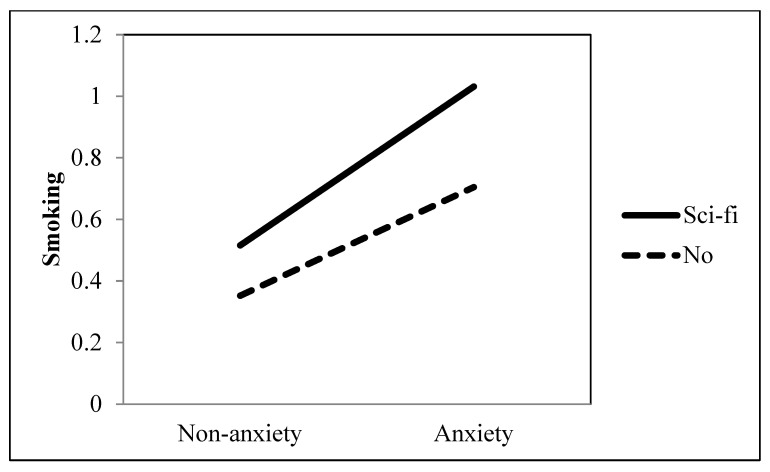
Interaction between anxiety and sci-fi as a TV genre on smoking behavior.

Research Question 1 investigated the role of ambiguously-valenced entertainment television viewing in the association between an anxiety disorder and smoking behavior. Our findings showed that the positive association between an anxiety disorder and smoking was significantly accentuated when they watched drama (*b* = 0.213, *p* = 0.002), music (*b* = 0.293, *p* < 0.001), and sci-fi (*b* = 0.170, *p* = 0.005) (see Table 1 and Figure 3, Figure 4 and Figure 5). However, movies did not moderate the association between an anxiety disorder and smoking (*b* = −0.129, *p* = 0.051).

### 3.4. The Moderating Roles of Informational Television Genres in the Association between an Anxiety Disorder and Smoking

**Figure 6:**
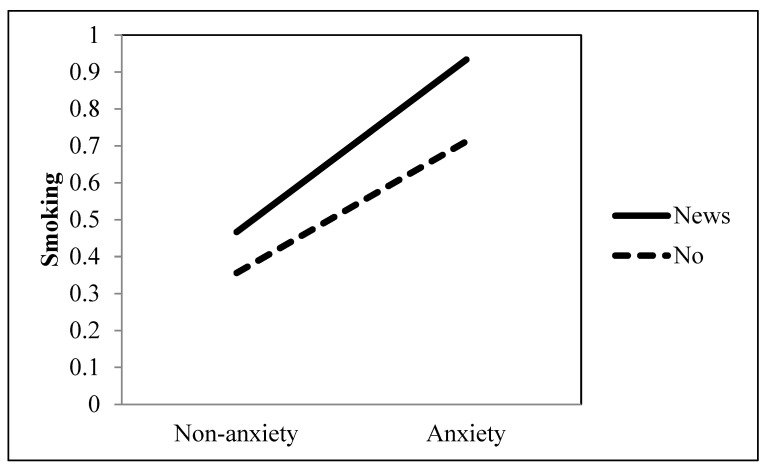
Interaction between anxiety and news as a TV genre on smoking behavior.

**Figure 7:**
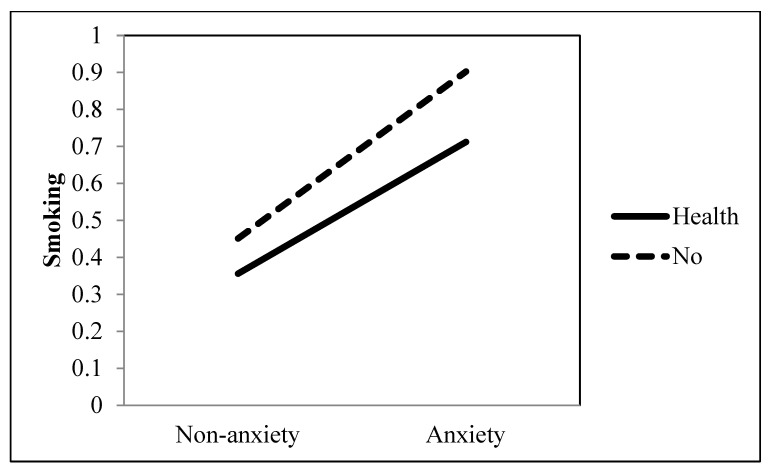
Interaction between anxiety and health information as a TV genre on smoking behavior.

Research Question 2 investigated the role of informational television viewing in the association between an anxiety disorder and the extent of smoking. Results indicated that the strength of the positive association between an anxiety disorder and the extent of smoking was significantly intensified when they watched television news (*b* = 0.124, *p* = 0.035) (see Table 1 and Figure 6). In contrast, the strength of the positive association between an anxiety disorder and the extent of smoking was significantly reduced when they watched health information programs (*b* = −0.183, *p* = 0.014) (see Table 1 and Figure 7). 

## 4. Discussion

### 4.1. Discussion

With the help of theoretical concepts from the mood management literature and a U.S. national survey data, we set out to examine the relationships among an anxiety disorder, television viewing, and smoking behavior. Our findings are critical for understanding the association between an anxiety disorder and smoking behavior as well as the conditional effects of television viewing on this relationship.

Our first results, showing that excitement-valenced entertainment television viewing (i.e., cartoons and sports) lessens the positive relationship between anxiety and smoking, are insightful. The positive effect of these entertainment programs that are considered to have uplifting and cheerful content supports the view of several earlier studies that entertainment viewing improves moods due to its hedonic-valenced content (e.g., [7,10,20,21]). Our findings not only support previous findings, but they also move beyond previous results by showing that television viewing of excitement-valenced entertainment programs could lower smoking behavior. Previous studies have elucidated the effects of media choice on mood alteration (e.g., [17,18,24]). Our results regarding behavioral effects provide insight such that specific types of entertainment content may have the power to alter not only individuals’ mood per se, but also their behaviors. 

Next, ambiguously-valenced television viewing such as drama, music, and sci-fi aggravates the positive relationship between anxiety and smoking. The capacity of our data is limited in unfolding the specific content in ambiguously-valenced television programs viewed (i.e., horror movie, sad music, comedy drama, etc.). However, we think, as the mood-congruence hypothesis assumes, that individuals tend to select media content that reflects, rather than deflects, their current moods [22]. In other words, anxious individuals might choose the content that is congruent with their existing state. Thus, we speculate that the exposure to this type of content may intensify their negative moods (e.g., [19]), and in turn, deteriorate behavioral responses. In addition, darker narratives [28] or risky behaviors that may be represented in many ambiguously-valenced television programs may allure risky behaviors. Likewise, sad music may dampen the spirits of anxious people, thereby providing a motivation to smoke, which can provide temporary relaxation for many individuals [29]. 

Finally, it is noteworthy that both news and health information programs moderate the positive relationship between anxiety and smoking behavior but in opposite directions. Specifically, although news viewing increases the association between anxiety and smoking, health-related information viewing decreases the relationship. The opposing findings between news and health information programs could be due to the content of each genre. Not surprisingly, health programs include informational content about health, which help viewers improve their knowledge and awareness of health risk [27]. On the other hand, news may have dysphoric content that is related to the stressful events in daily lives [7]. Our findings of the contradicting roles of two informational programs show the importance of differentiating informational television programs into news and health information programs. 

### 4.2. Limitations and Future Research

As with all research, our study comes with some limitations. Although the present study provides an insight into the categorization of entertainment genres by differentiating between excitement- and ambiguously-valenced programs, more specified categorization for the ambiguously-valenced entertainment genre might be needed. By doing so, future studies could clarify whether it is positively- or negatively-valenced entertainment content that influences behavior alteration. Additionally, although the present study provides evidence that specific television genres (i.e., excitement-valenced entertainment genre and health information genre) attenuate the likelihood that anxious individuals are involved in smoking behavior, it is beyond the scope of the present study to examine the underlying mechanisms of these relationships. Although our measurement of anxiety captures whether respondents are diagnosed with an anxiety disorder by a doctor, self-reports could be less accurate than clinical data obtained from medical records. Future studies could identify anxiety disorder patients with their health records to ascertain if the present findings hold. Finally, our dataset is from 2013, which means the data is a few years old. The media landscape has indeed changed since 2013; however, the television genres we measure remain consistent. 

### 4.3. Practical Implications

Patients with an anxiety disorder may need to refrain from viewing ambiguity-valenced entertainment television programs that especially feature sadness, suffering, and pain. In real life, it is possible that patients with an anxiety disorder lean toward dramatic movies that focus on emotional struggles. Given that individuals in a sad mood gravitate to serious films and crime dramas [23] and to dramatic movies with serious themes of death and discrimination [22], patients with mental illnesses are likely to lean toward negatively-valenced entertainment content. However, our findings suggest that dramatic plots, which consist of content in ambiguity-valenced entertainment programs, may accentuate their engagement in risky behaviors. Thus, despite the possible discrepancy between their preferences and recommendation, it might be best to opt out of ambiguity-valenced entertainment television programs. 

Clinicians, practitioners, and public health officials who aim to prevent patients with mental illnesses from engaging in risky behaviors can use these findings from our study to develop and implement educational intervention that focuses on patients’ everyday life. Given that people are less likely to report their mental health concerns to their healthcare professionals than physical ailments, educational strategies that help patients with mental illnesses to stay healthy and refrain from risky behavior need to be easily implemented at their home, rather than hospital settings. Our findings imply that public health officials can develop strategies using excitement-valenced entertainment television programs and health informational television programs in order to protect patients from risky behaviors. 

## 5. Conclusions

Despite some of these limitations, the current study used a unique national U.S. dataset to extend the mood management theory to understand risky behavior among patients with an anxiety disorder. Moreover, we differentiated entertainment television programing into two different categories to study the nuances of television viewing and its impact. The CDC has consistently warned us that smoking is the leading cause of preventable deaths in the U.S., amounting to 480,000 deaths every year [1]. We also know that anxiety disorders increase this risky behavior. Examining the various television genres that mitigate or augment this association is fundamental to understanding the potential factors that can help patients with anxiety disorders refrain from engaging in smoking behavior. 

## Figures and Tables

**Table 1:** Hierarchical regression analysis assessing the moderating effects of television genres on the association between an anxiety disorder and smoking behavior.

Variable Items	Smoking	
	*b*	*p*
Control variables		
Age	−0.044	0.000 ***
Gender (1 = male, 2 = female)	−0.016	0.287
Race (1 = white, 0 = others)	0.094	0.000 ***
Education	−0.091	0.000 ***
Income	−0.045	0.000 ***
Total hours per week spent watching television Main effects	0.018	0.000 ***
Anxiety	0.210	0.002 **
Excitement-valenced entertainment television genre		
Comedy	0.026	0.084
Cartoon	0.019	0.375
Sports	0.005	0.725
Ambiguously-valenced entertainment television genre		
Drama	−0.043	0.032 *
Movie	0.037	0.016 *
Music	−0.028	0.099
Sci-fi	0.106	0.000 ***
Informational television genre		
News	−0.001	0.946
Health	−0.116	0.000 ***
Interaction effects		
Anxiety and excitement-valenced entertainment genre		
Anxiety × Comedy	0.090	0.104
Anxiety × Cartoon	−0.170	0.009 **
Anxiety × Sports	−0.124	0.025 *
Anxiety and ambiguously-valenced entertainment genre		
Anxiety × Drama	0.213	0.002 **
Anxiety × Movie	−0.129	0.051
Anxiety × Music	0.293	0.000 ***
Anxiety × Sci-fi	0.170	0.005 **
Anxiety and informational television genre		
Anxiety × News	0.124	0.035 *
Anxiety × Health	−0.183	0.014 *
N	18,720	
Total R^2^	8%	

* *p* < 0.05, ** *p* < 0.01, *** *p* < 0.001.

## Data Availability

Data can be available upon request.
